# BVDV utilizes PGC-1α downregulation to remodel the mitochondrial metabolic microenvironment, enhancing viral replication and impairing host immunity

**DOI:** 10.3389/fvets.2026.1799048

**Published:** 2026-05-19

**Authors:** Hongming Zhou, Yixing Zhao, Yuxin Kong, Jiying Yin, Ning He, Qi Wang, Jiayin Liu, Tongbo Zhou, Yu Han, Shouyi Dong, Yaru Zhao, Naichao Diao, Kun Shi, Rui Du

**Affiliations:** 1 College of Animal Science and Technology, Jilin Agricultural University, Changchun, Jilin, China; 2 Agriculture College of Yanbian University, Yanji, China; 3 Liao Yuan Animal Disease Control Center, Liaoyuan, China; 4 College of Chinese Medicinal Materials, Jilin Agricultural University, Changchun, Jilin, China

**Keywords:** BVDV, mitochondrial quality control, PGC-1α, mitochondrial biogenesis, viral replication

## Abstract

**Introduction:**

Bovine viral diarrhea virus (BVDV) is a major pathogen affecting global livestock production, and virus-induced mitochondrial remodeling is closely associated with viral replication. However, the role of PGC-1α-mediated mitochondrial quality control in cytopathic BVDV strain NADL infection remains unclear.

**Methods:**

MDBK cells were infected with CP BVDV(NADL) to establish an in vitro infection model. Mitochondrial morphology, function, mitophagy levels, and the expression of related proteins were examined using transmission electron microscopy, fluorescence staining, confocal microscopy, Western blotting, and the pADV-CMV-FH-cox8-EGFP-mCherry vector dual-fluorescence system. PGC-1α expression was manipulated by plasmid-mediated overexpression or shRNA-mediated knockdown.Viral replication was quantified by qRT-PCR, and IFN-β secretion was assessed by ELISA.

**Results:**

CP BVDV(NADL) infection caused mitochondrial structural damage and dysfunction, accompanied by persistent downregulation of PGC-1α and its downstream target TFAM. Meanwhile, Drp1 expression was increased, shifting mitochondrial dynamics toward excessive fission. CP BVDV(NADL) infection also markedly enhanced PINK1-mediated mitophagy. Functionally, PGC-1α overexpression restored mitochondrial homeostasis, inhibited PINK1-dependent mitophagy, reduced IFN-β expression, and ultimately suppressed CP BVDV(NADL) replication. Conversely, PGC-1α interference further promoted mitophagy and increased mPTP opening.

**Discussion:**

These findings demonstrate for the first time that CP BVDV(NADL) promotes viral replication by manipulating PGC-1α-mediated mitochondrial quality control. This mechanism reveals a novel metabolic strategy used by BVDV and provides potential therapeutic targets for controlling CP BVDV(NADL) infection.

## 1 Introduction

Bovine viral diarrhea virus is an important member of the Pestivirus genus in the Flaviviridae family (1) and is the pathogen that causes bovine viral diarrhea disease (BVD) (2, 3). The virus is widely distributed globally and can infect ungulates such as cattle, deer, goats, and pigs, causing a range of clinical symptoms including diarrhea, abortion, and immunosuppression, leading to substantial economic losses in the global livestock industry and posing a severe challenge to wildlife conservation. The study found that NCP BVDV can induce mitophagy to suppress innate immune responses, and that mitochondria-mediated ferroptosis can promote BVDV-induced inflammatory responses *in vitro* (4). Dihydroartemisinin can exert anti-BVDV effects via mitochondria (5). Currently, the pathogenic mechanisms of BVDV in host cells, especially the molecular mechanisms underlying its interactions with host mitochondria, remain incompletely understood and warrant further investigation.

Mitochondria are essential organelles in eukaryotic cells that sustain cellular life, and their morphology, function, and abundance are tightly regulated by quality-control mechanisms (Mitochondrial quality control, MQC) (6). MQC mechanisms include mitochondrial biogenesis, fusion, fission, and mitophagy, and are essential for maintaining cellular health, adapting to physiological changes, and responding to various stresses (7). In recent years, studies have found that viral infection can alter the host cell's MQC network (8) and exploit these changes to promote viral replication and evade host immune responses. For example, Zika virus (ZIKV) perturbs MQC during placental cell infection, thereby suppressing host antiviral responses (9); Dengue virus (DENV) infection disrupts MQC and triggers inflammation (10); The gE protein of varicella-zoster virus (VZV) disrupts mitochondrial dynamics by triggering mitochondrial fission (11); Coronaviruses also evade innate immunity by altering mitochondrial dynamics and targeting the MAVS signaling pathway (12, 13). These studies underscore the central role of MQC in virus-host interactions; however, there are no detailed reports on whether and how CP BVDV(NADL) infection modulates host cell MQC.

Mitochondrial biogenesis is an important component of MQC (14) and involves the replication and expression of the mitochondrial genome, as well as the import of nuclear-encoded proteins into mitochondria, thereby ensuring the balance of mitochondrial number and function within the cell (15). Related studies indicate that viruses can exploit mitochondrial biogenesis to antagonize innate antiviral immunity (16). Peroxisome proliferator-activated receptor gamma coactivator-1α (PGC-1α) is a key regulator of mitochondrial biogenesis (17). It also serves as a hub linking mitochondrial dynamics, mitophagy, and other mechanisms. Studies have shown that PGC-1α can alleviate oxidative damage (18), reduce mitophagy (19), and regulate mitochondrial quality control (20). PGC-1α also plays a key role in viral infection (21). For example, in Epstein-Barr virus (EBV) infection, co-activation with STAT3 mediates immune evasion and has been shown to promote hepatitis B virus (HBV) replication (22), SIRT1 regulates mitochondrial biogenesis via the PGC-1α-TFAM pathway and mitigates mitochondrial damage caused by prions (23); the PGC-1α/ERRα axis also plays a role in human papillomavirus (HPV) E6 (24). Given the central role of PGC-1α in mitochondrial function and mitochondrial quality control (MQC) (25), as well as its demonstrated regulatory roles in other viral infections, whether PGC-1α participates in the CP BVDV(NADL) infection process and the specific mechanisms involved remain unknown.

This study aims to investigate dynamic changes in host cell mitochondrial quality control (MQC) following CP BVDV(NADL) infection and to elucidate how PGC-1α regulates MQC, modulates host immune responses, and governs viral replication. By elucidating the molecular mechanisms by which CP BVDV(NADL) manipulates mitochondrial homeostasis, this study provides new strategies and potential targets for the prevention and treatment of CP BVDV(NADL) infection.

## 2 Materials and methods

### 2.1 Cells, viruses, and plasmids

MDBK cells (Madin-Darby bovine kidney cells) were provided by the China Center for Type Culture Collection (CCTCC, Wuhan, China) and cultured in Dulbecco's Modified Eagle Medium (DMEM) supplemented with 10% fetal bovine serum (FBS from Lanzhou Rongye Company) in a humidified incubator at 37 °C with 5% CO_2_. CP BVDV NADL (NCBI reference sequence: NC_001461.1) is stored in the laboratory. pCAGGS (P0165) from MiaoLing Plasmid Platform (Wuhan, China). The adenoviral vector pADV-CMV-FH-cox8-EGFP-mCherry was provided by WZ Biosciences Inc, and all recombinant vectors used in this study were prepared in our laboratory.

### 2.2 Antibodies

We used the following primary antibodies:Anti-GAPDH(Invitrogen, PA5-85074); Anti-BetaActin(AC026), Anti-Mfn2, Anti-NRF2(A21176), Anti-PGC1α(A20995), Anti-VDAC1(A15735), Anti-Drp1(A13699), Anti-TFAM(A13552), Anti-Phospho-DRP1-S616(AP1353), Anti-TOMM20(A18047), Anti- LC3B(A19665), Anti-PINK1(A25301), Anti-P62(A19700), (The above antibodies are from ABclonal).

We used the following secondary antibodies: Goat Anti-Rabbit IgG H&L/HRP (Bioss).

### 2.3 Mitochondrial membrane potential measurement

Using JC-1 (Solarbio) to observe changes in mitochondrial membrane potential, MDBK cells infected with CP BVDV(NADL) (MOI = 1) for 48 h were washed twice with PBS, DMEM medium was added for later use, and the JC-1 staining working solution was added. Incubate at 37 °C for 20 min. After incubation, wash with staining buffer, add cell culture medium, and observe under a fluorescence microscope (Leica DMi8).

### 2.4 Transmission electron microscopy observation of mitochondrial morphology changes

After MDBK cells were infected with CP BVDV(NADL) (MOI = 1) for 48 h. Discard the medium, wash the cells three times with PBS, scrape them from the flask walls, and resuspend them. Transfer the cell suspension to a 1.5 mL centrifuge tube, centrifuge at 4 °C at 4,000 rpm to pellet the cells, remove PBS, add 2.5% glutaraldehyde at room temperature and fix for 1 h, then transfer to a 4 °C refrigerator to fix for 24 h. Wash with PBS, fix with 1% osmium tetroxide for 1 h, then wash with PBS again, sequentially dehydrate in 30%, 50%, 70%, 80%, 95%, and 100% ethanol, followed by infiltration and embedding for ultrathin sectioning. Stain with uranyl acetate for 25 min, rinse with water, stain with lead citrate for 7 min, and observe using a transmission electron microscope (TEM, Hitachi HT-7800) at 80 kV.

### 2.5 Detection of Reactive Oxygen Species (ROS) Production

MDBK cells were seeded at a density of 2 × 10^5^ cells/well in a 48-well plate. After infecting MDBK cells with CP BVDV NADL (MOI = 1) for 24 h, samples were collected. ROS levels in the cells were detected using a ROS detection kit (Beyotime). DCFH-DA was diluted in serum-free medium, Remove the medium, add ROS staining working solution, and incubate at 37 °C for 30 min. Wash cells three times with PBS, add DMEM medium, and observe ROS production under a fluorescence microscope (Leica DMi8).

### 2.6 ATP production level measurement

Seed MDBK cells at a density of 2 × 10^5^ cells per well in a 6-well plate, infect with CP BVDV(NADL) (MOI = 1), and collect samples at various time post-infection. Using the ATP assay kit (Beyotime) according to the manufacturer's instructions, extract ATP, then detect with a fluorescence microplate reader and calculate the ATP content.

### 2.7 Mitochondrial network observation

Seed 2 × 10^5^ MDBK cells onto glass coverslips in a 48-well plate and infect with CP BVDV(NADL) (MOI = 1). At the specified time post-infection, stain MDBK cells with 200 nm MitoTracker Red CMXRos (Beyotime, C1035), then apply an antifade reagent (Orileaf) to cover the cells, and mount the coverslips onto glass slides. Observe with a laser confocal microscope (Leica, STELLARIS5, Germany).

### 2.8 Mitochondrial isolation

Isolate mitochondria using a mitochondrial isolation kit (Beyotime). After infection of MDBK cells with CP BVDV(NADL) (MOI = 1) for 24, 36, and 48 h, remove the medium, digest the cells with trypsin, centrifuge at 1,000 rpm for 10 min, resuspend the cells in pre-chilled PBS, centrifuge at 4 °C at 3,000 rpm for 5 min, add pre-chilled mitochondrial lysis buffer (incubate on ice for 15 min). Transfer the suspension into a glass homogenizer and grind repeatedly. Centrifuge at 4 °C, 3,000 rpm for 10 min; gently remove the supernatant at 4 °C, then centrifuge at 14,000 rpm for 10 min to pellet mitochondria. The pellet is the mitochondrial extract, and the supernatant is the cytoplasmic extract, which will be used for subsequent Western blot and qPCR experiments.

### 2.9 Analysis of mitochondrial DNA expression in the cytoplasm

Extract mtDNA from the cytoplasmic suspension obtained in the previous step using a DNA extraction kit (Solarbio). Use qPCR to compare the relative levels of mitochondrial DNA to nuclear DNA to assess mt- DNA. The expression level of mitochondrial DNA is determined by mt-ND1(26); GAPDH serves as an internal control. The primers used were:

GAPDH F:TTCAACGGCACAGTCAAGGCA;GAPDH R:CCACCACATACTCAGCAGC

ND1 F:ACCCTCGATTTCGCTATGACCAAC;ND1 R:TGTTTGTGGTGGGATGCCTGATTG.

### 2.10 Interference and overexpression

The short hairpin RNA (shRNA) targeting PGC-1α (target site: GGTGCAGTGACCAATCAGAAA) was designed and synthesized by GenePharma. pCAGGS-PGC-1α was designed based on the GenBank-registered bovine PGC-1α gene sequence (Gene ID: 338446), with primers amplifying the homologous arms designed at the XhoI restriction site of the pCAGGS vector. The primers were synthesized by Sangon Company, the fragment was obtained, and the fragment was ligated into the pCAGGS vector. Using Lipofectamine 3,000 reagent to transfect the vector, the treated cells were cultured in DMEM supplemented with 10% FBS for 24 h to obtain MDBK cells targeted by sh-PGC-1α and MDBK cells expressing pCAGGS-PGC-1α, after which they were infected with CP BVDV(NADL) (MOI = 1) for 48 h, and the cells were harvested for Western blot analysis and other experiments.

### 2.11 Western blot analysis

Cells subjected to different treatments were harvested separately, and total cellular protein was extracted using a protein extraction kit (Bestbio). Protein concentrations were quantified separately using a BCA assay kit (Beyotime), and protein samples were prepared. Run the gel at 150 V, select a PVDF membrane with an appropriate pore size for the target protein's molecular weight, and perform the protein transfer at a constant current of 400 mA. Block the PVDF membrane by immersing it in 5% skim milk powder in TBST for 2 h. Remove the blocking solution, incubate the PVDF membrane with the primary antibody solution, and refrigerate at 4 °C for 12 h. The primary antibody solution was recovered and stored. The membrane was washed with TBST buffer, then incubated with a secondary antibody working solution prepared in TBST at room temperature for 2 h. Omit the secondary antibody; wash with TBST three times, each for 10 min. After development with the ECL chemiluminescent detection reagent in the dark, capture the luminescent signal with an imaging system and analyze the results.

### 2.12 Mitophagy detection

Use the pADV-CMV-FH-cox8-EGFP-mCherry vector to monitor mitophagy (27). Inoculate 5 × 10^4^ MDBK cells into a culture dish containing a cell-covered slide, then infect with the pADV-CMV-FH-cox8-EGFP-mCherry adenovirus. After different treatments, MDBK cells were infected with CP BVDV(NADL) (MOI = 1) for 48 h. Observations were performed using a laser scanning confocal microscope (Leica STELLARIS 5, Germany).

### 2.13 Detection of mPTP opening

Following cellular stress, the mitochondrial permeability transition pore (mPTP) opens, facilitating the release of mitochondrial contents. It serves as a crucial pathway for the exchange of various mitochondrial components with the cytoplasm, reflecting mitochondrial function and status (28). Seed MDBK cells at a density of 2 × 10^5^ cells per well in a 48-well plate and infect with CP BVDV(NADL) (MOI = 1) for 48 h. Using 10 μm CCCP as a positive control, mitochondria in cells were treated with Calcein AM and CoCl_2_ from the mPTP detection kit (Beyotime). Observations were performed using a confocal microscope (Leica, STELLARIS5, Germany).

### 2.14 ELISA

Plate layout using a 6-well plate with a plating density of 2 × 10^5^ cells per well and 50% confluence. Cells are transfected with an overexpression plasmid and with shRNA. Follow the Lipofectamine 3,000 protocol for dosing in 6-well plates. Overexpression plasmid (PGC-1α) at 2.5 μl per well and shRNA (PGC-1α) at 5 μl per well. Experiments are performed in triplicate, with a corresponding blank control group, infection group CP BVDV(NADL) (MOI = 1), infection with overexpression plasmid and shRNA, and infection with overexpression plasmid and shRNA. After transfection, replace the medium and inoculate with CP BVDV(NADL) at 24 h; for the control group, replace with culture medium. After an additional 24 h of culture, collect the supernatant using sterile tubes. Centrifuge at 4 °C for 20 min at 2000 g, collect the supernatant. Read the absorbance (OD value) of each well at a wavelength of 450 nm on a microplate reader.

### 2.15 Virus replication level detection

Detect CP BVDV(NADL) 5'-UTR expression levels by qRT-PCR (29). RNA was extracted using the RNA extraction kit (CWBIO), and cDNA was prepared using PrimeScript™ RT (Takara). The obtained cDNA was mixed with TB Green (Takara) and analyzed using the qTOWER^3G^ real-time quantitative PCR instrument (Analytik Jena AG); relative expression levels of the target genes were standardized to GAPDH as the internal control. The primer sequences are as follows:

GAPDH-F:TTCAACGGCACAGTCAAGGCA;GAPDH-R:CCACCACATACTCAGCAGC

5' UTR-F:GRAGTCGTCARTGGTTCGAC; 5'UTR-R:TCAACTCCATGTGCCATGTAC

### 2.16 Apoptosis detection

Seed MDBK cells into a 48-well plate at a density of 2 × 10^5^ cells per well, infect with CP BVDV (NADL) (MOI = 1) for 48 h, and use CCCP (10 μm) as a positive control. The cells were then stained sequentially with Annexin V-FITC and propidium iodide (Beyotime, C1062S). The cells were incubated at room temperature in the dark for 30 min, followed by observation under a fluorescence microscope (Leica DMi8).

### 2.17 CCK-8

Seed MDBK cells into a 96-well plate at a density of 2 × 10^4^ cells per well, infect with CP BVDV (NADL) (MOI = 1) for 48 h. Cell viability was determined by measuring absorbance at 450 nm using the Cell Counting Kit-8 (Beyotime, C0037).

### 2.18 LDH release detection

Seed MDBK cells into a 96-well plate at a density of 2 × 10^4^ cells per well, infect with CP BVDV (NADL) (MOI = 1) for 48 hours, use CCCP (10 μm) as a positive control, and inhibit autophagy with bafilomycin A1(50 nm) and apoptosis with Z-VAD-FMK (30 μm). Follow the instructions for the LDH Cytotoxicity Assay Kit (Beyotime, C0016) to measure absorbance at 450 nm and assess cell death.

### 2.19 Statistical analysis

All data are expressed as mean ± standard deviation (mean ± S.D.), and were analyzed using one-way ANOVA with Bonferroni *post hoc* tests. Then Tukey multiple-comparison tests were conducted using GraphPad Prism 8.0 (GraphPad Software Inc., San Diego, CA, USA). ^*^P < 0.05; ^**^P < 0.01; ^***^P < 0.001; ns: not significant.

## 3 Results

### 3.1 BVDV infection causes mitochondrial structural damage and leads to mitochondrial dysfunction

**Figure 1:**
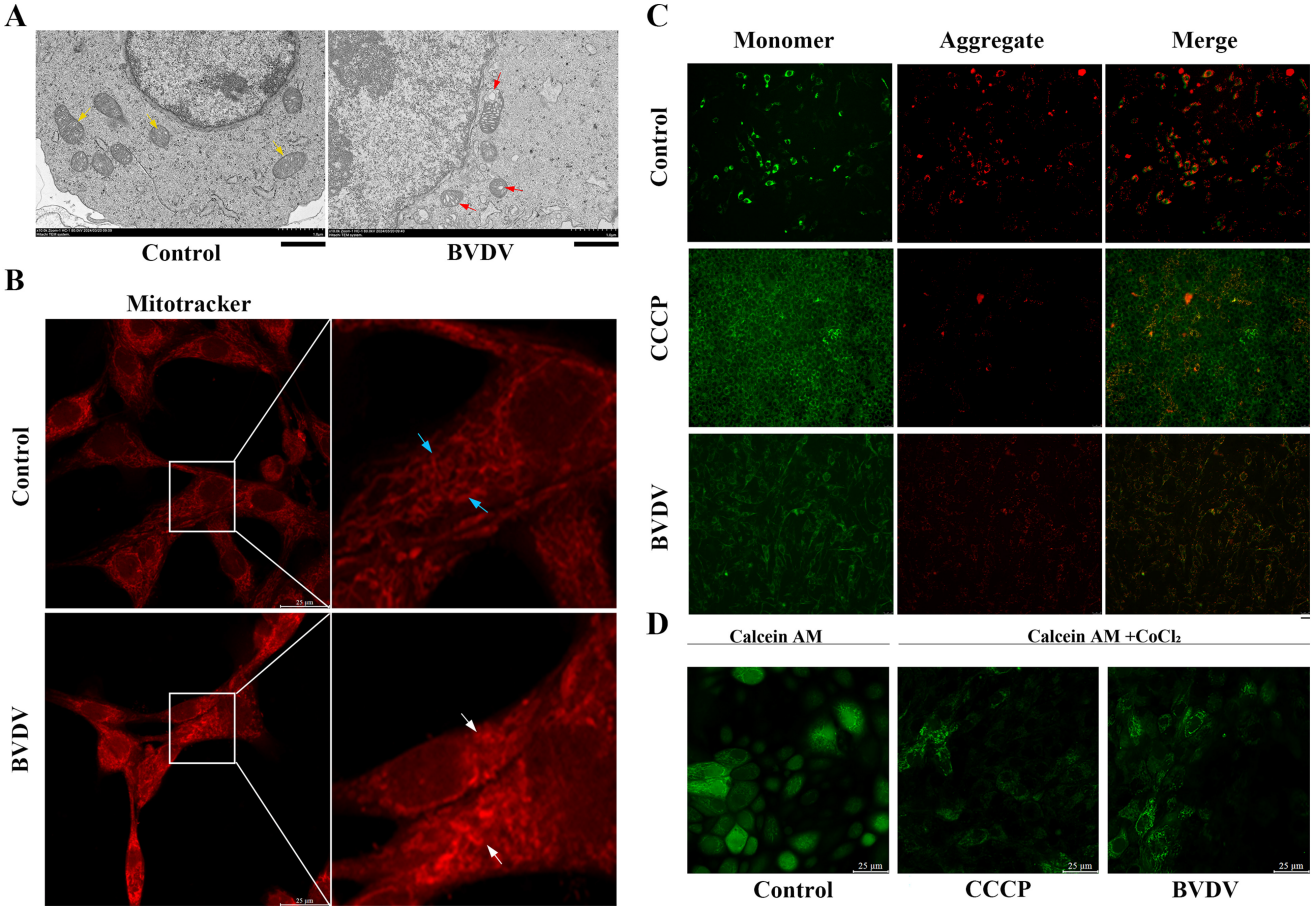
BVDV infection causes mitochondrial structural damage and leads to mitochondrial dysfunction. Result **(A)** Transmission electron microscopy observing mitochondrial morphology in BVDV-infected cells (scale bar: 1 μm), hours after infection:48 hpi(Healthy mitochondria: yellow arrow; Damaged mitochondria: red arrows); Result **(B)** MDBK cell mitochondrial network labeled by itotracker (scale bar: 25 μm), hours after infection:48 hpi(Healthy mitochondrial network: blue arrows; Damaged mitochondrial network: white arrow); Result **(C)** changes in mitochondrial membrane potential in BVDV-infected cells (scale bar: 250 μm), hours after infection:48 hpi; Result **(D)** opening level of mitochondrial membrane permeability transition pore after BVDV infection (scale bar: 25 μm), hours after infection:48 hpi.

After CP BVDV(NADL) infection, the host cell mitochondria undergo significant changes in both morphology and function. To investigate whether CP BVDV(NADL) infection affects the morphology and function of host cell mitochondria, the changes in mitochondrial structure and function were analyzed after MDBK cells were infected with CP BVDV(NADL). Transmission electron microscopy results showed that, compared with the control group, mitochondrial ultrastructure in infected cells underwent significant changes, including rupture of the outer mitochondrial membrane, swelling and rupture of cristae, and a significant loss of mitochondrial structural integrity (Figure 1A). Subsequently, MitoTracker was used to fluorescently label the mitochondrial network, and confocal microscopy was employed to observe mitochondrial dynamics. The results showed that in normal cells, mitochondria form a continuous reticular network, whereas after CP BVDV(NADL) infection, the mitochondrial network became significantly fragmented and displayed a punctate distribution (Figure 1B). To further evaluate mitochondrial function, JC 1 was used to detect mitochondrial membrane potential. The results showed that the negative control cells predominantly exhibited red fluorescence, indicating higher mitochondrial membrane potential; in contrast, the CP BVDV(NADL)-infected group and the CCCP-positive control group displayed similar fluorescence characteristics, with enhanced green fluorescence and reduced red fluorescence, indicating a significant decrease in mitochondrial membrane potential (Figure 1C). Finally, Calcein AM staining was used to assess the opening status of the mPTP. Results showed that compared with the control group, cytoplasmic fluorescence intensity was significantly reduced in both the CP BVDV(NADL)-infected and positive control groups, indicating a marked increase in the extent of mPTP opening (Figure 1D).

### 3.2 BVDV infection causes mitochondrial energy metabolism disorder and induces oxidative stress

**Figure 2:**
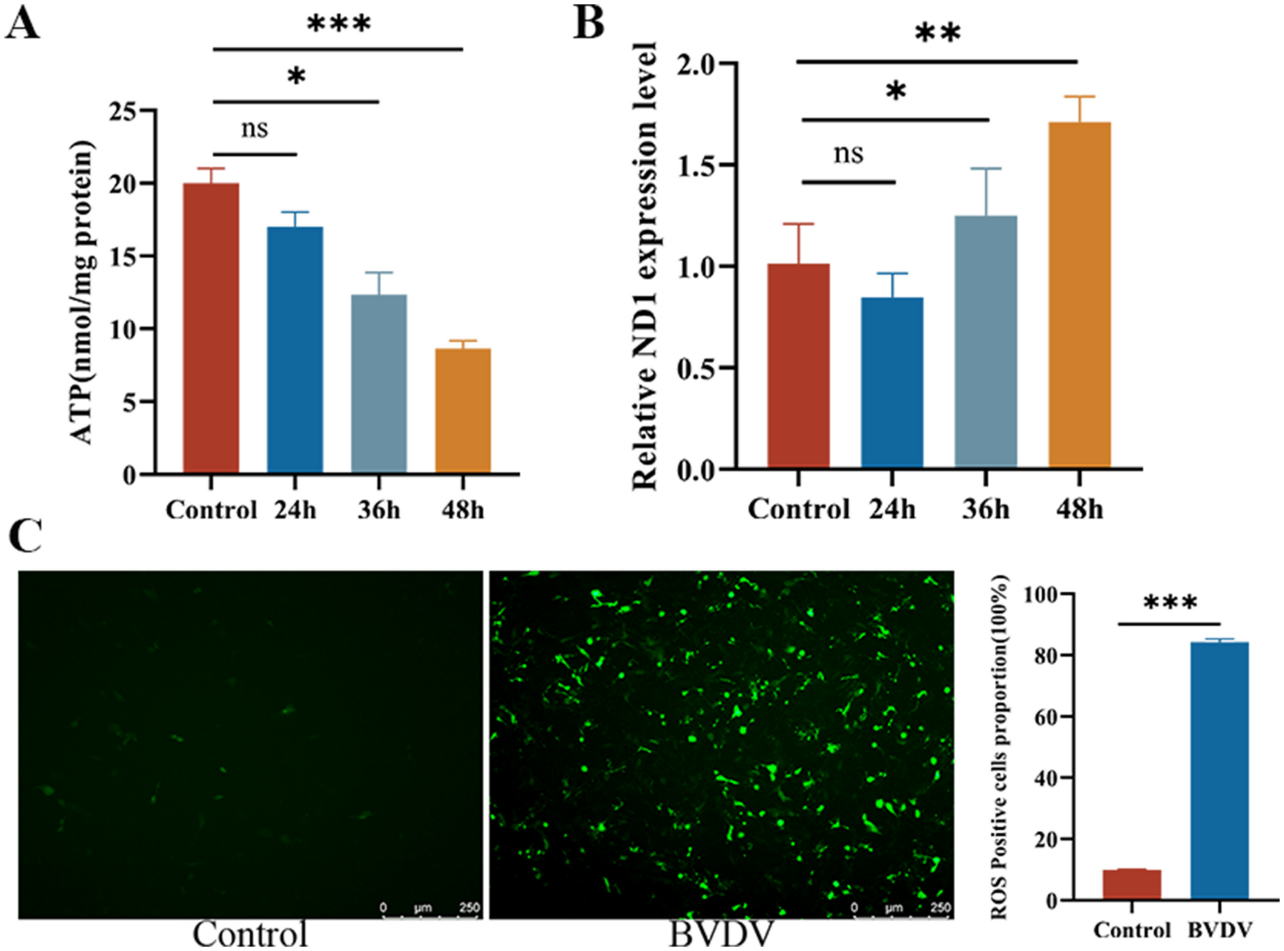
BVDV infection causes mitochondrial energy metabolism disorders and induces oxidative stress. Result **(A)** Changes in cellular ATP content at different time points after BVDV infection (*n* = 3), hours after infection: 24,36,48 hpi; Data represented mean ± S.E. ^*^P < 0.05; ^***^P < 0.001; ns: not significant compared with control groups. Result **(B)** Changes in mt-ND1 expression at different time points after BVDV infection (*n* = 3), hours after infection: 24,36,48 hpi; Data represented mean ± S.E. ^*^P < 0.05; ^**^P < 0.01; ns: not significant compared with control groups. Result **(C)** Observation of ROS generation after BVDV infection (scale bar: 250 μm) and analysis of fluorescence intensity, hours after infection: 24 hpi; Data represented mean ± S.E. ^***^P < 0.001 compared with control groups.

CP BVDV(NADL) infection inhibits mitochondrial energy metabolism in a time-dependent manner and induces oxidative stress and mitochondrial damage. To evaluate the impact of CP BVDV(NADL) infection on the physiological function of host cell mitochondria, ATP production in MDBK cells was measured at different time points after infection (24, 36, and 48 h). The results showed that, compared with the control group, ATP levels in CP BVDV(NADL)-infected cells declined significantly with increasing infection time, indicating inhibition of mitochondrial energy metabolism (Figure 2A). In addition, to further assess the extent of mitochondrial damage and the cytosolic release of mitochondrial DNA, the cytosolic fraction was separated, and qPCR was used to detect changes in mt-ND1. The results showed that the copy number of MT-ND1 in the cytosol gradually increased with longer CP BVDV(NADL) infection time, indicating that CP BVDV(NADL) infection induces mitochondrial damage and promotes mtDNA translocation from mitochondria to the cytosol (Figure 2B). Using fluorescent probes to detect reactive oxygen species (ROS) production in cells after CP BVDV(NADL) infection, the green fluorescence intensity in the CP BVDV(NADL)-infected group was significantly enhanced compared with the control group, indicating a markedly increased ROS level (Figure 2C).

### 3.3 BVDV infection induces mitochondrial dynamics imbalance and activates mitophagy

**Figure 3:**
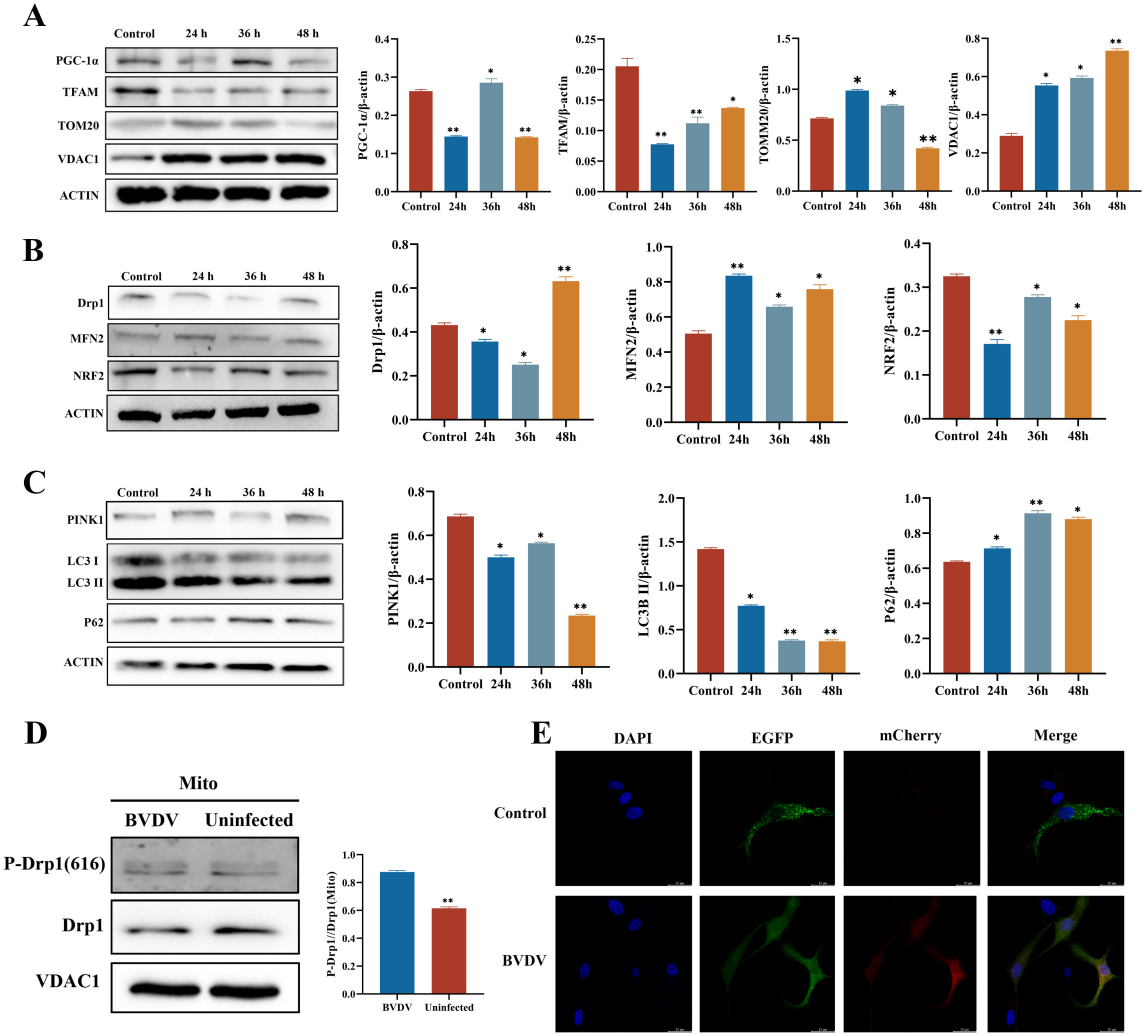
BVDV infection induces mitochondrial dynamics imbalance and activates mitophagy. Result **(A)** Changes over time in mitochondrial biogenesis and mitochondrial membrane-related genes and proteins after BVDV infection and analysis, hours after infection: 24,36,48 hpi; Data represented mean ± S.E. ^*^P < 0.05; ^**^P < 0.01 compared with control groups. Result **(B)** Changes over time in genes and proteins related to mitochondrial dynamics after BVDV infection and analysis, hours after infection: 24,36,48 hpi; Data represented mean ± S.E. ^*^P < 0.05; ^**^P < 0.01 compared with control groups. Result **(C)** Changes over time in genes and proteins related to mitophagy after BVDV infection and analysis, hours after infection:24,36,48 hpi; Data represented mean ± S.E. ^*^P < 0.05; ^**^P < 0.01 compared with control groups. Result **(D)** Expression and analysis of p-Drp1(S616) in mitochondria after BVDV infection, hours after infection: 48 hpi; Result **(E)** Mitophagy flux after BVDV infection (scale bar: 25 μm), hours after infection: 48 hpi; Data represented mean ± S.E. ^**^P < 0.01 compared with BVDV groups.

CP BVDV(NADL) infection significantly alters the expression patterns of mitochondrial biogenesis, dynamics, and mitophagy-related factors, suggesting that mitochondrial quality control processes are disrupted. To determine whether CP BVDV(NADL) infection affects mitochondrial quality control processes in host cells, MDBK cells were infected with CP BVDV(NADL), and the expression changes of key mitochondrial quality control factors were detected at different infection time points. The results show that, compared with the negative control, the expression level of the mitochondrial biogenesis-related factor PGC-1α after CP BVDV(NADL) infection first decreases with the infection time, then briefly increases, and finally decreases again; TFAM expression levels continuously decrease as the infection time prolongs. The expression of the outer mitochondrial membrane protein TOMM20 increases in the early stage of infection and then decreases; VDAC1 levels rise overall after CP BVDV(NADL) infection and do not show significant changes with infection time (Figure 3A). Further assessment of expression changes in mitochondrial dynamics–related proteins. The results show that compared with the control group, the expression level of the mitochondrial fission protein Drp1 after CP BVDV(NADL) infection increases significantly with the extension of infection time; the expression levels of the mitochondrial fusion protein MFN2 and the antioxidant stress factor NRF2 also show a trend of increasing with longer infection time (Figure 3B). In terms of mitophagy-related proteins, compared with the control group, PINK1 protein expression gradually increases with infection time in the CP BVDV(NADL)-infected group, while LC3-II protein expression shows a decreasing trend, and P62 protein expression increases significantly with extended infection time (Figure 3C). To further evaluate the activation state of mitochondrial fission, the expression levels of p-Drp1 (Ser616) in the cytosolic and mitochondrial fractions were measured 48 h after CP BVDV(NADL) infection. The results show that p-Drp1 (Ser616) protein expression is significantly higher in the CP BVDV(NADL)-infected group than in the control group (Figure 3D). In addition, mitochondrial double fluorescent labeling was performed using an adenoviral vector pADV-CMV-FH-cox8-EGFP-mCherry, and mitochondrial autophagy levels after CP BVDV(NADL) infection were observed by confocal microscopy. Double fluorescence labeling of mitochondria was performed, and confocal microscopy was used to assess mitochondrial autophagy levels after CP BVDV(NADL) infection. The results show that, compared with the control group, the red fluorescence signal is significantly enhanced while the green fluorescence signal is weakened in the CP BVDV(NADL)-infected group, indicating a marked increase in mitochondrial autophagy levels (Figure 3E).

### 3.4 PGC-1α participates in the regulation of mitochondrial quality control under BVDV infection

**Figure 4:**
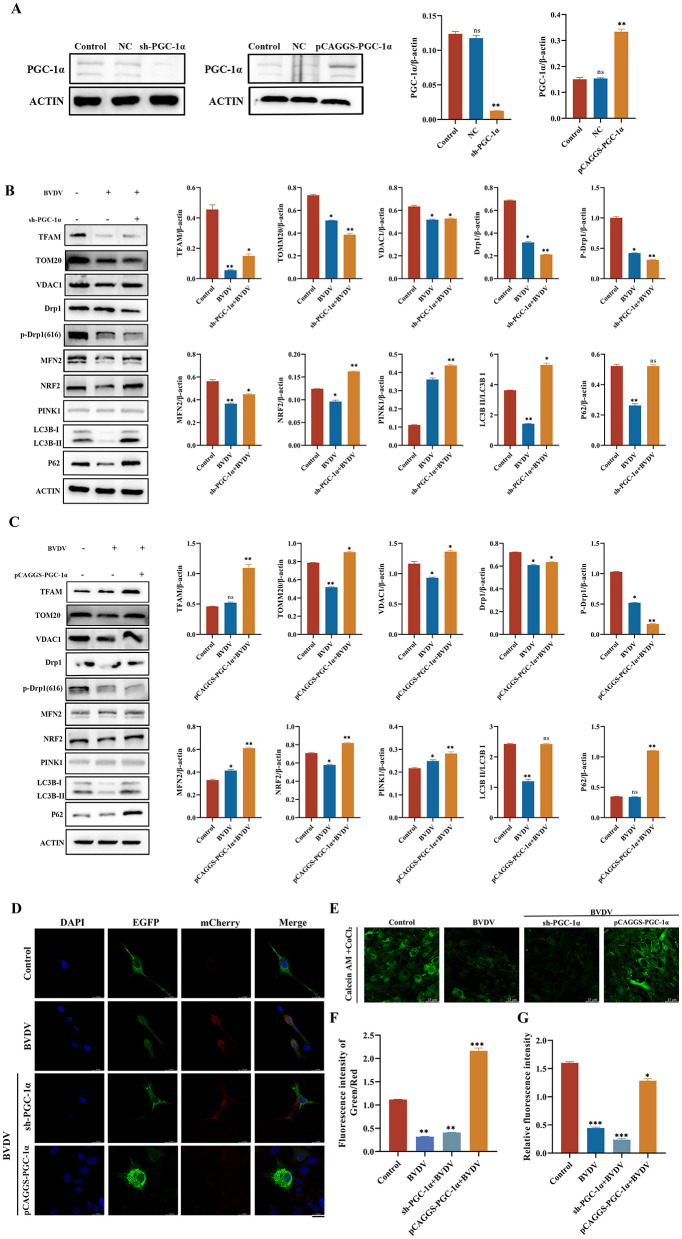
PGC-1α involvement in the regulation of mitochondrial quality control under BVDV infection. Result **(A)** suppression and overexpression levels of the PGC-1α gene, hours after infection: 24 hpi; Data represented mean ± S.E. ^**^P < 0.01; ns: not significant compared with control groups. Result **(B)** changes in mitochondrial quality control-related genes after BVDV infection when PGC-1α gene expression is interfered with, hours after infection: 24 hpi; Data represented mean ± S.E. ^*^P < 0.05; ^**^P < 0.01; ns: not significant compared with control groups. Result **(C)** changes in mitochondrial quality control-related genes after BVDV infection when PGC-1α gene is overexpressed, hours after infection: 24 hpi; Data represented mean ± S.E. ^*^P < 0.05; ^**^P < 0.01; ns: not significant compared with control groups. Result **(D, F)** after interference and overexpression of the PGC-1α gene, changes in mitophagy and fluorescence intensity after BVDV infection (scale bar: 25 μm), hours after infection: 24 hpi; Data represented mean ± S.E. ^**^P < 0.01; ^***^P < 0.001 compared with control groups. Result **(E, G)** after interference and overexpression of the PGC-1α gene, detection of mPTP and fluorescence intensity after BVDV infection (scale bar: 25 μm), hours after infection: 24 hpi; Data represented mean ± S.E. ^*^P < 0.05; ^***^P < 0.001; compared with control groups.

Modulating PGC-1α expression significantly alters mitochondrial biogenesis, dynamics, and mitophagy-related phenotypes under CP BVDV(NADL) infection. The previous results show that CP BVDV(NADL) infection significantly disrupts host mitochondrial quality control. To further clarify the role of the key regulator of mitochondrial biogenesis, PGC-1α, in this process, models overexpressing PGC-1α and PGC-1α knockdown by shRNA were constructed. The bovine PGC-1α gene was cloned into the pCAGGS mammalian expression vector and transfected into MDBK cells to achieve overexpression, while shRNA was used to knock down PGC-1α expression; the construction and intervention efficiency were validated (Figure 4A). First, assess changes in mitochondrial biogenesis-related factors following CP BVDV(NADL) infection, with PGC-1α expression as a regulated factor. The results show that after perturbing PGC-1α expression, the mitochondrial transcription factor TFAM is significantly downregulated, while TOMM20 and VDAC1 show no significant changes; after overexpressing PGC-1α, TFAM expression is significantly upregulated, and TOMM20 and VDAC1 still exhibit no obvious changes (Figure 4B). Subsequently, analyze the expression changes of proteins related to mitochondrial dynamics. The results show that after perturbing PGC-1α expression, the expression levels of the mitochondrial fission protein Drp1 and the fusion protein MFN2 are significantly downregulated, while the antioxidant stress factor NRF2 is significantly upregulated, and the phosphorylation level of Drp1 at Ser616 is markedly increased. On the contrary, after overexpressing PGC-1α, the expression levels of Drp1, MFN2, and NRF2 are all significantly increased, while the phosphorylation level of Drp1 at Ser616 is markedly decreased (Figure 4C). Further examination of changes in autophagy-related mitochondrial proteins. After silencing PGC-1α, the expression levels of PINK1 and p62 are significantly upregulated, and the conversion of LC3 from type I to type II is enhanced; after overexpressing PGC-1α, PINK1 and p62 expression levels are markedly reduced, and LC3-II levels also decrease correspondingly (Figure 4C). To further evaluate the regulatory effect of PGC-1α on mitophagy, an adenoviral vector pADV-CMV-FH-cox8-EGFP-mCherry was used to achieve dual fluorescent labeling of mitochondria, followed by infection with CP BVDV(NADL) after regulating PGC-1α expression. Confocal results show that, compared with the control group, silencing PGC-1α significantly promotes mitophagy, while overexpressing PGC-1α markedly inhibits mitophagy levels (Figures 4D, F). Additionally, by assessing mPTP opening, it was found that silencing PGC-1α significantly increased mPTP opening (Figures 4E, G).

### 3.5 PGC-1α regulates mitochondrial function, immune responses, and viral replication levels under BVDV infection conditions

**Figure 5:**
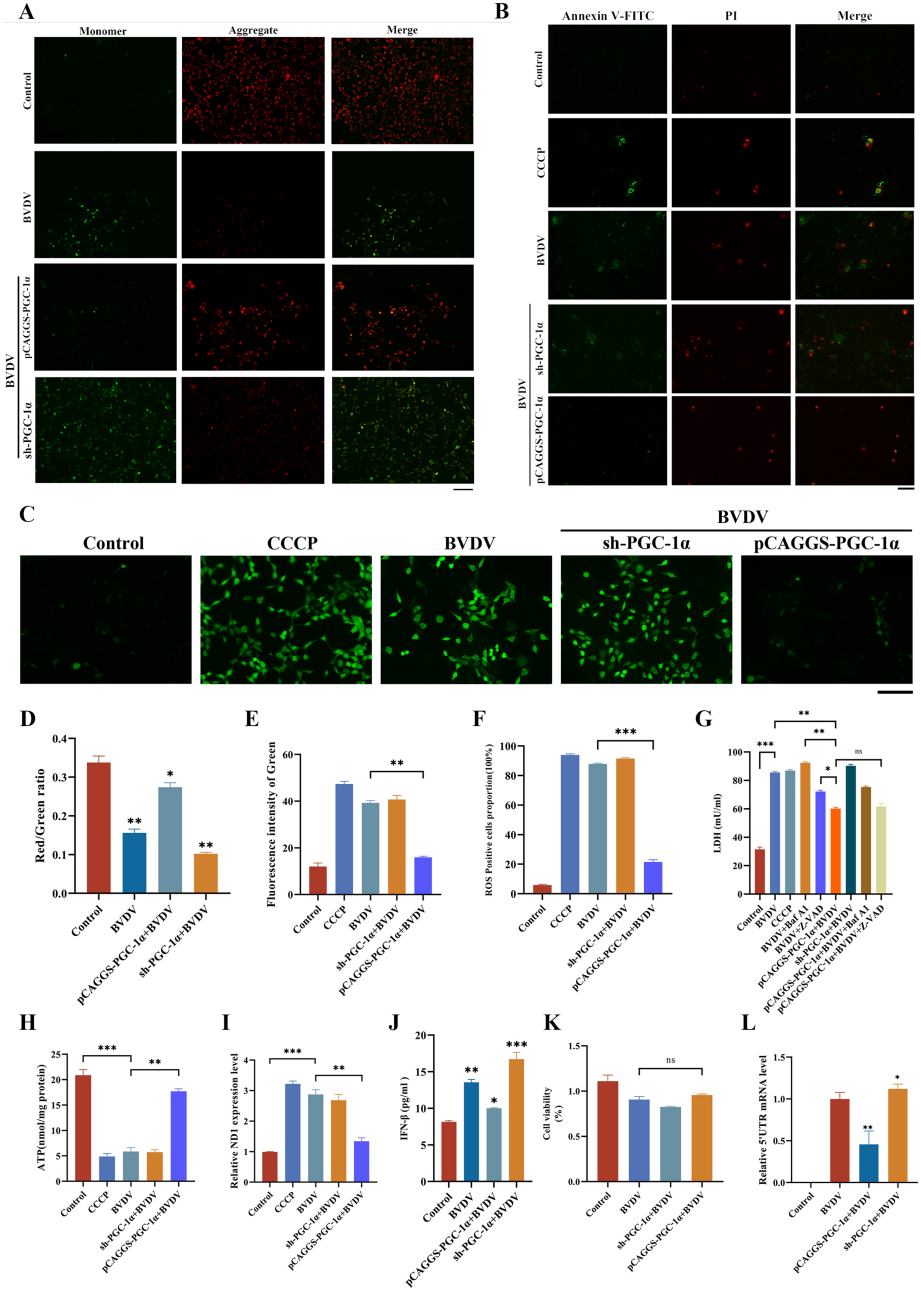
PGC-1α regulation of mitochondrial function, immune response, and viral replication levels under BVDV infection. Result **(A, D)** analysis of mitochondrial membrane potential changes and fluorescence intensity after interference and overexpression of the PGC-1α gene in BVDV-infected cells (scale bar: 250 μm), hours after infection: 24 hpi; Data represented mean ± S.E. ^*^P < 0.05; ^**^P < 0.01 compared with control groups. Result **(B, E)** analysis of apoptosis status after interference and overexpression of the PGC-1α gene in BVDV-infected cells (scale bar: 250 μm), hours after infection: 24 hpi; Data represented mean ± S.E. ^**^P < 0.01 compared with BVDV groups. Result **(C, F)** analysis of ROS production after interference and overexpression of the PGC-1α gene in BVDV-infected cells (scale bar: 250 μm), hours after infection: 24 hpi; Data represented mean ± S.E. ^***^P < 0.001 compared with BVDV groups. Result **(G)** analysis of LDH production after interference and overexpression of the PGC-1α gene in BVDV-infected cells (*n* = 3), hours after infection: 24 hpi; Data represented mean ± S.E. ^***^P < 0.001, comparison of the BVDV groups with the control groups. ^**^P < 0.01, comparison of the pCAGGS-PGC-1α + BVDV groups with the BVDV groups and comparison of the pCAGGS-PGC-1α + BVDV groups with the BVDV + Baf A1 groups. ^*^P < 0.05, comparison of the pCAGGS-PGC-1α + BVDV groups with the BVDV + Z-VAD groups. ns: not significant, comparison of the pCAGGS-PGC-1α + BVDV groups with the pCAGGS-PGC-1α + BVDV + Z-VAD groups. Result **(H)** analysis of ATP production after interference and overexpression of the PGC-1α gene in BVDV-infected cells (*n* = 3), hours after infection: 24 hpi; Data represented mean ± S.E. ^***^P < 0.001, comparison of the BVDV groups with the control groups. ^**^P < 0.01, comparison of the pCAGGS-PGC-1α + BVDV groups with the BVDV groups. Result **(I)** changes in ND1 gene expression after interference and overexpression of the PGC-1α gene in BVDV-infected cells (*n* = 3), hours after infection: 24 hpi; Data represented mean ± S.E. ^***^P < 0.001, comparison of the BVDV groups with the control groups. ^**^P < 0.01, comparison of the pCAGGS-PGC-1α + BVDV groups with the BVDV groups. Result **(J)** analysis of IFN-β expression after interference and overexpression of the PGC-1α gene in BVDV-infected cells (*n* = 3), hours after infection: 24 hpi; Data represented mean ± S.E. ^*^P < 0.05; ^**^P < 0.01; ^***^P < 0.001 compared with control groups. Result **(K)** analysis of cell viability after interference and overexpression of the PGC-1α gene in BVDV-infected cells (*n* = 3), hours after infection: 24 hpi; Data represented mean ± S.E. ns: not significant, comparison of the pCAGGS-PGC-1α + BVDV groups with the BVDV groups. Result **(L)** expression level of the BVDV 5βUTR gene (*n* = 3), hours after infection: 24 hpi. Data represented mean ± S.E. ^*^P < 0.05; ^**^P < 0.01 compared with BVDV groups.

Overexpression of PGC-1α can ameliorate mitochondrial dysfunction associated with CP BVDV(NADL) infection, accompanied by changes in host immune responses and viral replication levels. PGC-1α participates in mitochondrial quality control during CP BVDV(NADL) infection, and a further assessment of its effects on mitochondrial function, host immune responses, and viral replication is warranted. First, we detected changes in mitochondrial membrane potential in cells infected with CP BVDV(NADL) after regulating PGC-1α expression. The results showed that overexpression of PGC-1α significantly restored the mitochondrial membrane potential decreased by CP BVDV(NADL) infection (Figures 5A, D). We detected apoptosis using Annexin V/PI. Compared with the CP BVDV (NADL) group, overexpression of PGC-1α reduced the level of apoptosis but did not alter the extent of necrosis (Figures 5B, E). ROS production was observed using fluorescence microscopy. The results showed that, compared with the CP BVDV (NADL) group, overexpression of PGC-1α reduced ROS production levels (Figures 5C, F). LDH (lactate dehydrogenase) assay results indicate that CP-type bovine viral diarrhea virus (NADL) infection leads to significant cellular injury. Overexpression of PGC-1α significantly inhibits excessive mitochondrial autophagy, markedly reduces LDH release, and alleviates cellular damage. Upon addition of the autophagy inhibitor bafilomycin A1, the mitochondrial autophagy process was blocked, the cytoprotective effect of PGC-1α was significantly weakened, whereas the apoptosis inhibitor Z-VAD-FMK had virtually no effect on the cytoprotective effect of PGC-1α (Figure 5G). Meanwhile, the results regarding ATP production indicate that, compared to the CP BVDV (NADL) group, overexpression of PGC-1α restored ATP production levels (Figure 5H). Subsequently, we assessed changes in mitochondrial DNA levels in the cytosol. qPCR results showed that, upon PGC-1α overexpression, the expression level of the mtDNA marker gene ND1 in CP BVDV(NADL) infected cells was significantly reduced (Figure 5I). Further analysis of the effect of PGC-1α on innate immune responses showed that, compared with the control group, overexpression of PGC-1α led to a significant decrease in IFN-β expression in CP BVDV(NADL)-infected cells (Figure 5J). To evaluate the regulatory role of PGC-1α in viral replication, we measured cell viability and the expression levels of the CP BVDV(NADL) 5′UTR after modulating PGC-1α expression. The results showed no significant differences in cell survival rates among the groups. This indicates that, compared to the CP BVDV(NADL) group, interfering with PGC-1α expression did not significantly affect viral replication levels. Conversely, PGC-1α overexpression significantly reduced the expression of the CP BVDV(NADL) 5′UTR, suggesting that viral replication was markedly inhibited (Figures 5K, L).

## 4 Discussion

Bovine viral diarrhea virus is a major pathogen that poses a serious threat to global livestock industries and wild ruminants, and its replication strategy within host cells and mechanisms of persistent infection remain to be elucidated in depth. This study systematically reveals how CP BVDV(NADL) infection drives changes in mitochondrial quality control (MQC) in the host by precisely modulating MQC pathways, particularly by suppressing the key transcriptional co-activator PGC-1α, thereby remodeling mitochondrial function, promoting viral replication, and impacting host immune responses.

Viral infection can alter mitochondrial dynamics, mediating mitochondrial apoptosis and altering mitochondrial metabolic state and intrinsic cellular immunity to sustain intracellular survival (30, 31). Related studies show that Hepatitis C virus (HCV) induces mitochondrial fission, increases reactive oxygen species (ROS), causes mitochondrial Ca^2+^ overload, and impairs oxidative phosphorylation (32). HBV and its HBx protein can cause mitochondrial morphological abnormalities and decreased mitochondrial membrane potential (33). Dengue virus (DENV) infection can trigger mitochondrial dynamics imbalance, elevated reactive oxygen species (ROS), release of mtDNA into the cytoplasm, and activation of innate immune pathways (34). Influenza A virus can directly enter mitochondria and induce a decrease in mitochondrial membrane potential, as well as accelerated mitochondrial fission (35). Varicella-zoster virus (VZV) infection can regulate mitophagy (11), VZV-induced mitochondrial dynamics imbalance is closely related to severe deterioration of mitochondrial health (36). Our study confirms, from both morphological and functional perspectives, the significant damage to MDBK cell mitochondria caused by CP BVDV(NADL) infection. Transmission electron microscopy and MitoTracker fluorescence labeling results show that mitochondrial ultrastructure integrity is compromised after infection, manifested as outer membrane rupture, cristae swelling, and mitochondrial network fragmentation. Functional analysis further confirms that CP BVDV(NADL) infection causes a significant decline in mitochondrial membrane potential (MMP), promotes abnormal opening of the mitochondrial permeability transition pore (mPTP), and triggers a sustained decrease in intracellular ATP levels, collectively revealing severe mitochondrial dysfunction. Additionally, CP BVDV(NADL) infection increases reactive oxygen species (ROS) levels and promotes the release of mitochondrial DNA (mtDNA) into the cytoplasm. Mitochondrial DNA (mtDNA), as a damage-associated molecular pattern (mtDAMP), can be released to activate the cGAS-STING signaling pathway and induce innate immune responses (37), analogous to mitochondrial damage and oxidative stress induced by dengue virus infection.

Mitochondrial dynamics is the processes of mitochondrial fusion and fission, are key to maintaining mitochondrial homeostasis (38). Related studies indicate that viral infection induces activation of Drp1 (39). Promote mitochondrial fission and block MAVS-mediated innate immune signaling (40). Related studies indicate that excessive mitochondrial fission (41) triggers mitochondrial dysfunction (42). This study found that CP BVDV(NADL) infection significantly upregulates the expression of the mitochondrial fission-related protein Drp1 and its phosphorylation at Ser616, accompanied by mitochondrial network fragmentation, indicating that CP BVDV(NADL) actively induces mitochondrial fission. Overactive mitochondrial fission not only generates small, damaged mitochondria and exacerbates dysfunction, but may also promote mtDNA release. Related studies indicate that PRV infection, by promoting Mfn-mediated mitochondrial fusion suppresses PRV replication (43). This study found that changes in the expression of the mitochondrial fusion protein Mfn2 and the overall downregulation of the antioxidant transcription factor Nrf2 further support the strategy that CP BVDV(NADL) exacerbates mitochondrial damage by disrupting mitochondrial dynamics balance and suppressing antioxidant stress responses.

Mitophagy (44) is an important MQC mechanism (45) for removing damaged mitochondria (46) and maintaining mitochondrial homeostasis. Related studies indicate that influenza A virus (IAV) infection can induce mitophagy (47). This study confirms that CP BVDV(NADL) infection markedly promotes selective mitophagy, as demonstrated by the mitochondrial autophagy dual-fluorescence system and elevated PINK1 protein expression. Although selective mitophagy is activated, overall autophagy (LC3 II level) shows a decreasing trend, and p62 protein expression is markedly increased. This phenomenon suggests that CP BVDV(NADL) may finely regulate the autophagy pathway, activating selective mitophagy to clear damaged mitochondria while inhibiting overall autophagy to prevent cell death caused by excessive autophagy. This strategy of “selective clearance with global suppression,” similar to ZIKV and coronaviruses (48), may help CP BVDV(NADL) maintain host cell viability by utilizing MQC mechanisms, thereby supporting ongoing viral replication. Studies show that NCP BVDV (NY 1) promotes mitochondrial fission and activates PINK1/Parkin-dependent mitophagy to clear damaged mitochondria: increased expression of PINK1/Parkin and recruitment of Parkin to mitochondria recruit autophagy-related molecules to the damaged mitochondria (1). Compared with the mechanism of NCP BVDV, this study infected MDBK cells with CP BVDV (NADL); in terms of the mitophagy phenotype, the Cox8-EGFP-mCherry mitochondrial dual-fluorescence probe showed increased red signal and decreased green signal, indicating that mitochondria were delivered to acidic lysosomes, with PINK1 levels increasing over the course of infection (49). CP BVDV (NADL) infection likewise triggers mitochondria-related stress signaling and induces mitophagy-related processes. Following CP BVDV (NADL) infection, mitophagy is activated, and mitochondria sequestration by lysosomes is enhanced, but whether it exhibits Parkin-dependent mitophagy similar to the NCP type remains to be further validated (4). This difference suggests that the two biotypes of BVDV may utilize mitochondrial quality control differently: the NCP type tends to maintain cell survival and immune silencing via Parkin-mediated mitophagy to support persistent infection. By contrast, CP BVDV (NADL) triggers mitophagy while autophagy markers show different changes, suggesting that the autophagy network may be altered and linked to its cytopathic phenotype. Existing studies also suggest that there may be commonalities in immune evasion: CP BVDV and NCP BVDV can both induce complete autophagy, and by activating the ROS–ER stress axis upregulate BECLN1, thereby leveraging the–MAVS interaction to promote MAVS degradation and suppress type I interferon responses (50). This indicates that both CP- and NCP-type BVDV can target the mitochondrial antiviral signaling platform MAVS via autophagy-related pathways, but this remains to be further validated in CP BVDV (NADL).

PGC-1α (51) acts as the core transcriptional coactivator of mitochondrial biogenesis and plays a key role in regulating mitochondrial function across various aspects of energy metabolism, cellular stress responses, and MQC (52), making it a crucial mediator (53). Related studies indicate that downregulation of PGC-1α can induce mitochondrial dysfunction (54). This study reports for the first time that CP BVDV(NADL) infection markedly suppresses the expression of PGC-1α and its downstream target TFAM (55). The downregulation of PGC-1α may exacerbate mitochondrial dysfunction by limiting the biogenesis of new mitochondria. More importantly, overexpression and interference experiments with PGC-1α confirm that it not only regulates mitochondrial biogenesis but also broadly participates in mitochondrial dynamics and mitophagy. Overexpression of PGC-1α effectively reverses the mitochondrial membrane potential decline caused by CP BVDV (NADL) infection, inhibits abnormal opening of the mitochondrial permeability transition pore (mPTP), and reduces the level of cytosolic mitochondrial DNA (ND1). It attenuates apoptosis, lowers ROS production, restores ATP generation, and decreases LDH production. LDH assays were used to quantify cell death, and, using apoptosis inhibitors (56, 57) and autophagy inhibitors as controls, we clarified the role of PGC-1α in mitophagy during CP BVDV (NADL) infection. CP BVDV (NADL) infection can significantly induce excessive mitophagy and cause evident cellular damage. Overexpression of PGC-1α can significantly suppress excessive mitophagy, markedly reduce LDH release, and alleviate cellular damage. After the autophagy flux inhibitor Baf A1 is added, the mitochondrial autophagy process is blocked, and the protective effect of PGC-1α on cells is significantly weakened, whereas the caspase inhibitor Z-VAD-FMK does not alter the protective effect of PGC-1α on cells. Moreover, in the absence of significant differences in cell viability, overexpression of PGC-1α markedly reduces CP BVDV (NADL) replication. PGC-1α may exert its protective effect mainly by inhibiting CP BVDV (NADL) induced excessive mitophagy, rather than relying primarily on intrinsic apoptotic pathways. These results collectively indicate that PGC-1α plays a central role in maintaining mitochondrial functional homeostasis.

Related studies indicate that PGC-1α mediates immune evasion in EBV infection by coactivating STAT3 (58), and it has been shown to promote HBV replication (59). SARS-CoV-2 hijacks host cell mitochondrial function to enhance viral fitness (60). In our study, overexpression of PGC-1α inhibited viral replication, indicating that its function varies across different viruses and host contexts. As a node in the mitochondrial regulatory network, its dominant direction may differ under various backgrounds, leading to differences in immune evasion and antiviral efficacy. Related studies show that EBV's LMP1 can stabilize PGC-1α, thereby regulating metabolism and immune networks, supporting immune evasion and activating tumor-associated pathways, indicating PGC-1α's direct role in virus-induced immune regulation (61). Viral infections downregulate PGC-1α, triggering mitochondrial dysfunction, altered ROS levels, and mtDNA release, highlighting the importance of PGC-1α as a key node in maintaining mitochondrial homeostasis in viral diseases (62). The PB1-F2 protein of Influenza A may target mitochondria via PGC-1α, thereby altering mitochondrial function and immune responses (63). This study found that PGC-1α overexpression significantly inhibits CP BVDV(NADL) replication while reducing IFN-β expression levels. Overexpression of PGC-1α, while improving abnormal mitochondrial function during BVDV infection, reduces the level of the cytosolic mtDNA marker gene ND1, and is accompanied by decreased IFN-β expression, with viral replication significantly reduced. This suggests that the inhibitory effect of PGC-1α overexpression on BVDV replication may not rely on upregulation of IFN-β expression. Coupled with the decrease in ND1, the reduction of IFN-β may reflect decreased mtDNA leakage after alleviation of mitochondrial damage, while innate immune activation driven by mtDAMP signaling is reduced. Some studies have indicated that innate immune transcription factors, such as IRF3/IRF7, can directly participate in the regulation of the PGC-1α promoter, thereby linking the immune transcription program with mitochondrial biogenesis and metabolic regulation (64, 65). Therefore, the relationship between PGC-1α and the IFN axis may be bidirectional under different infection contexts. We speculate that PGC-1α may primarily act by maintaining mitochondrial homeostasis and improving cellular energy metabolism and oxidative stress milieu, thereby weakening the intracellular conditions required for viral replication, resulting in a scenario distinct from IFN-β (66). In this study, the causal relationship between the type I IFN pathway and changes in viral replication has not been directly validated using approaches such as exogenous IFN-β supplementation, blockade of the IFNAR–JAK–STAT signaling (67), or assessment of downstream ISGs; further validation is needed in follow-up studies. Combining the known role of PGC-1α in regulating cellular metabolic environments and maintaining mitochondrial functional homeostasis. We hypothesize that PGC-1α may generate a non–interferon–dependent antiviral effect by remodeling cellular energy supply (68, 69), regulating key metabolic pathways, or enhancing cellular antioxidant capacity (70, 71), thereby disrupting the metabolic conditions on which viral replication depends (72). Future studies should further investigate the specific metabolic pathways and molecular mechanisms by which PGC-1α regulates CP BVDV(NADL) replication.

In summary, this study systematically reveals that CP BVDV(NADL) infection disrupts the host mitochondrial quality control system by suppressing PGC-1α, regulating mitochondrial fission and selective autophagy, thereby inducing severe mitochondrial dysfunction and ultimately promoting viral replication. This study first identifies PGC-1α as a crucial node connecting mitochondrial homeostasis regulation to CP BVDV(NADL) replication, providing a new perspective on CP BVDV(NADL) pathogenic mechanisms and offering strong theoretical justification and potential therapeutic strategies for interventions targeting the mitochondrial quality control system to disrupt persistent CP BVDV(NADL) infection.

## Data Availability

The original contributions presented in the study are included in the article/supplementary material, further inquiries can be directed to the corresponding author/s.
